# A qualitative study exploring medication management in people with dementia living in the community and the potential role of the community pharmacist

**DOI:** 10.1111/hex.12534

**Published:** 2017-01-19

**Authors:** Ian D. Maidment, Lydia Aston, Tiago Moutela, Chris G. Fox, Andrea Hilton

**Affiliations:** ^1^ Pharmacy School of Life and Health Sciences Aston University Birmingham UK; ^2^ School of Life and Health Sciences Aston University Birmingham UK; ^3^ University of East Anglia Norwich UK; ^4^ University of Hull Hull UK

**Keywords:** community pharmacists, dementia, informal carers, medication management

## Abstract

**Background:**

The prevalence of dementia is increasing rapidly. People with dementia may be prescribed complex medication regimens, which may be challenging for them and any carers involved to safely manage.

**Objective:**

To describe and understand the key challenges, in relation to medication issues, experienced by people with dementia and their informal carers dwelling in the community and the potential role of community pharmacists.

**Design:**

Qualitative semi‐structured interviews.

**Participants:**

People with dementia, informal carers and health and social care professionals (HSCPs).

**Results:**

Thirty‐one participants (eleven informal carers, four people with dementia and sixteen HSCPs) were interviewed. Three key themes were identified: the key challenges, improving medication management and the role of pharmacists. The caring role commonly included responsibility for medication management which created both practical problems and an emotional burden. This burden was worsened by any difficulty in obtaining support and if the person with dementia was on a complex regimen. Participants believed that the process could be improved by coordinated and on‐going support from HSCPs, which should focus on the informal carer. Medication reviews, particularly when conducted in the home environment, could be helpful.

**Conclusion:**

Medication management for people with dementia living in the community is a complex process, and informal carers have a key role, which they frequently find challenging. Community pharmacists could have an enhanced role in this area, but would need to work within a more multidisciplinary environment outside the pharmacy.

## 1 INTRODUCTION

It is estimated that dementia has a global prevalence of 47.5 million individuals.^1^ In the UK, 800 000 people live with dementia – this figure is expected to double by 2040 reflecting global predictions.^1^, ^2^ The presence of dementia potentially increases the likelihood of the presence for risk factors for adverse drug reactions such as lack of pharmacy input, drug interactions, comorbidity and polypharmacy, amongst others.^3^, ^4^, ^5^, ^6^ The cognitive impairment characteristic of dementia may result in a lack of capacity to safely self‐administer medication.^7^, ^8^ Despite people with dementia being particularly susceptible to adverse drug events, they are commonly prescribed complex medication regimes and on average receive five different medicines.^7^, ^9^

Medication management “is the entire way that medicines are selected, procured, delivered, prescribed, administered and reviewed to optimize the contribution that medicines make to producing informed and desired outcomes of patient care”.^10^ With increasing cognitive impairment, the core symptom of dementia, medication management is often shifted from the personal responsibility of the person with dementia to the control of formal (paid) or informal (family) carers particularly with complex regimens.^11^, ^12^, ^13^, ^14^, ^15^ Informal carers often have a key role in ensuring safe and effective medication use as they may conduct up to 10 medication management activities every day, including awareness of and managing side‐effects, and decisions to administer medication.^16^, ^17^ Previous research has identified the role of informal carers as advocates and their potential contribution to patient safety.^18^ Unlike health professionals, family and friends who are informal carers may not receive any training or have access to evidence‐based information to appropriately support medication management.^17^, ^19^ In fact, informal carers may not feel equipped for such an augmented role which they can find burdensome and stressful and this in turn may affect the informal carer's and the person with dementia's quality of life.^20^, ^21^, ^22^, ^23^, ^24^

Without the support and collaboration of health professionals involved, informal carers may struggle to adequately support medication management especially of complex regimes.^8^ However, some health‐care professionals lack time and awareness of the administration practicalities and the realities of informal carers and people with dementia.^25^, ^26^ Furthermore, the information about medication regimens, provided by health‐care professionals, can be complex and sometimes is not directly communicated to the informal carer due to the restriction of confidential information.^17^, ^19^, ^26^

The role of community pharmacists is changing from being focused on the supply of medicines to providing clinical services.^27^, ^28^ These services are focused on helping patients obtain the optimal benefit from prescribed medication by advising other clinicians and working directly with patients and others.^29^, ^30^, ^31^, ^32^ The change in role is supported by national and international policy.^29^, ^33^ The available literature argues that community pharmacy services available for people with dementia are poorly developed and the need for further research on ways to optimize medication management in people with dementia living in the community has been identified.^25^, ^34^

It is important then to explore the perspectives of the key stakeholders, including informal carers and people with dementia, to understand how community pharmacists can support people with dementia living in the community with safe medication management. The objectives of the study were to describe and understand the key challenges, in relation to medication issues, experienced by people with dementia and their informal carers dwelling in the community, and the potential role of community pharmacists.

## 2 METHODS

### 2.1 Design

An exploratory qualitative study design that followed Consolidated Criteria for Reporting Qualitative studies (COREQ) guidelines^35^ was employed (see appendix for further details). A focus group conducted at the Alzheimer's Society and involving informal carers had previously identified the need for research in this area.^26^

### 2.2 Participants and recruitment

Participants were recruited from the Alzheimer's Society, Dementia UK, local General Practice (GP) surgeries, professional networks and local dementia support groups (including a support group for people with dementia from the Black and Minority Ethnic [BME] community in Yorkshire). The institutions were contacted by LA and IM via telephone and email and were enquired about their willingness to collaborate in advertising the study. Recruitment was also conducted through snowball effect as contacts were requested to suggest other people with dementia, informal carers and HSCPs who might be willing to take part in this study.

### 2.3 Data collection

Semi‐structured face‐to‐face interviews were conducted. Two interview guides were used: one for people with dementia and their informal carers and one for HSCPs (see appendix for further details). This was the selected type of interview as it gives participants the opportunity to expand their answers freely and to provide in‐depth reflections about their lived experiences. Furthermore, it allowed obtaining data that were suitable for conducting a Framework analysis. On three occasions, both the informal carer and the person with dementia were together in the room during the interview. This allowed the person with dementia to provide further insight into what the informal carer was saying. Each interview lasted between 30 and 60 minutes. Interpreters from the same community were used for all the interviews with informal carers from the BME community.

### 2.4 Inclusion criteria

Informal carers were eligible if:

- They had provided or still provide some sort of assistance with medication management to a person who has been diagnosed with dementia and is living in the community;
- Did not receive any sort of payment (excluding receipt of carers’ allowance).

HSCPs were eligible if:

- They had been in contact with or had been providing assistance to people with dementia

### 2.5 Data analysis

A qualitative framework analysis was undertaken in order to explore the experiences and perspectives of the participants. Framework analysis, which has been specifically designed for applied qualitative research that commences deductively from specified aims and objectives, was used to organize and make sense of the data.^36^, ^37^ NVIVO software was used to manage the data.

The transcripts were transcribed verbatim. TM and IM independently reviewed the transcripts. Disagreements on the interpretation and analysis of the data were then discussed between TM and IM. Any disagreements or differences in interpretation of the data between TM and IM were resolved, as necessary, with discussions involving the wider team (with AH the final arbiter) until consensus was achieved. A systematic cross‐comparison analysis was undertaken by TM, and reviewed by IM to identify the similarities and differences between the different participants and to develop a set of themes which represent the whole corpus of data. TM and IM then discussed and agreed the final structure of the matrix for the analysis.

## 3 RESULTS

Thirty‐one participants, eleven informal carers, four people with dementia, sixteen HSCPs (four GPs, five nurses, three social care professionals [paid formal carers] and four community pharmacists), were interviewed. Three main themes were identified. Key challenges experienced by informal carers and people with dementia (the caring role, the challenges of the condition), improving medication management in people with dementia (empowerment and communication from health professionals) and the role of pharmacists.

### 3.1 Key challenges experienced by informal carers and people with dementia: the caring role, the challenges of the condition

Interviews with informal carers and people with dementia provided information on the challenges that they experience in relation to medication management. Nearly half of the carers reported that they had no problem in terms of the practicalities of managing the medication. Although it is a complex process, by adopting an organized routine, carers reported that the practical aspects of managing medication did not have any impact on their lives:

So in your opinion, does managing your medication affect your daily life (question from research associate)?

Not really, no, not really. I've got it sussed out. (Informal carer; pp3).

No, not really, no. It's all right if I do it in advance (discussing organising the medication), you see, I know what to do, so… It's really three times a day I have to remember. But then it's usually when we're eating, so it's part of the ritual with the food. (Informal carer; pp4).

Some (but not all) informal carers stated that compliance packs could help them organize the medication:

Before this sort of thing came out which I didn't know about, they came in a small bottle and, obviously, you have to take certain ones at certain times. Well, I used to get into a right two and eight (Cockney rhyming slang for a state). And it…well, it just was a muddle. And then (community pharmacy chain), I used to go to (community pharmacy chain), and then they started putting them into packs like this. (Informal carer; pp2).

However, there is still frequently an emotional burden associated with medication management and carers expressed an obligation to being responsible for managing the medication of the person they are looking after; this included prioritizing the health of the person they cared for over their own health:

Sometimes I feel fed up but what can I do? That is my duty…I forget my medicine but I never forget his (Informal carer; pp27).

On further exploration, informal carers linked this burden to the fact that the medication was not improving the behaviours and cognitive problems characteristic of dementia:

I was frustrated with myself. Why? With the medicine and she was getting worse and worse…the way she was behaving that I was worried about. (Informal carer – pp25).

Ensuring adherence to medication may be difficult. Due to increasing cognitive impairment, the person with dementia may not understand the need for medication. Again, the difficulties proliferate as the complexity of the regimen increases, potentially making the person with dementia very dependent on the carer:

I wouldn't know which ones to take, there's too many of them. (Person with dementia; pp1).

I have missed (doses of medication) occasionally. (Person with dementia; pp11.)

According to some informal carers, their caring duty was shown to be particularly challenging when medication needed to be taken at different times of the day:

So all these [health issues] require medication…It's keeping them separate that is the real problem (Informal carer; pp14).

Moreover, keeping track of the supply of medication was also highlighted as being a potential difficulty:

Make sure that you got enough tablets to last (Informal carer; pp3).

I think the main problem with the medication was having to go and get it and remembering, “Oh, there's only that many left of that particular one, I'd better order some more.” And I seemed to be up and down there twice a week. (Informal carer; pp4)

The impact that taking care of the medication of a person with dementia has on the carer may be even more evident when they are forced to make decisions about administering medication without sufficient support from HSCPs:

And now I discovered we've run out of brown (Warfarin) and they didn't bother to give as a prescription for it and it was the weekend. And I was going to go to the doctor but what I did was I've got a pill cutter. (OK). I cut the blue into two, I hope that's the correct dosage, I don't know. (Informal carer; pp14).

Lack of access to doctors was seen as a barrier to effective medication management. Clinicians’ lack of time to address carers’ or patients’ concerns was flagged by several informal carers:

This is what I feel I need to see Dr X, I really do. I feel that the tablets that she's on, they are not doing anything. I often wonder, sort of, to experiment and not give her any tablets at all for a week and see what the outcome would be. But then it might be dangerous. It could be you know, she could just fall off the planet. This is why I want to speak to him. But it's like trying to see the Pope. (Informal carer; pp2).

Thus, informal carers often need to decide whether or not to follow instructions, or change the regimen without seeking advice:

[Doctor said] Every three days, but I don't give it to her every three days because it's a morphine patch, it's for pain. She isn't saying that she's…any pain. Following the prescription she'd be taking 8 paracetamol a day which I think is far too much to be honest with you. (Informal carer – pp16).

### 3.2 Improving medication management in people with dementia: empowerment and communication from health professionals

All groups (people with dementia, informal carers and HSCPs) identified that people with dementia and their informal carers needed more support with medication management. Medication management is frequently solely dependent on informal carers, and therefore, targeting them is the best way to improve the process:

I find organising things in the short term, I find it difficult (Person with dementia; pp1).

So that's where the help needs to be improved, empowering carers…You can't empower the patients because they're already losing them (to the symptoms of dementia). (GP; pp5).

Yet, informal carers may have their own health problems and therefore have to manage the medication of two people: themselves and the person they care for. The majority of the informal carers agreed that having to manage both regimens can be difficult as there is:

A lot to think about. Yes. That's why I like to think about and with (the person with dementia cared for) not being on the ball, sort of business…I have to think about as well. (Informal carer ‐ pp3).

HSCPs need to provide clear explanations so that informal carers fully understand the treatments in order to decrease the risk of medication errors:

If they've sat there in a consultation and me or anyone else…”Oh, they've completely got that” and then a few months later…although the drug is no longer on…their medical records, at home they've still got boxes of tablets and they're still taking them and they shouldn't be (GP; pp15)

Therefore, HSCPs should supply:

A little bit more…explanation…and then it's easier to take that in and we'll keep that in our memories’. (Informal carer; pp1)

Furthermore, informal carers believed that medication management could be improved if HSCPs:

Listen to the concerns of the relatives (Informal carer; pp14)

It was identified by a HSCP that written communication in addition to verbal could be used as a way to help ensure that informal carers and people with dementia understand their treatment:

Written instructions, pictures and making sure that their…family understand what to do. (Nurse; pp9)

However, this topic needs to be addressed carefully as the information needs to be tailored to person with dementia and their informal carer. It is important to not only provide the right instructions for the right illness but make sure that the person has the ability to understand the message (as the earlier quote from the GP pp15 also highlighted):

It's crazy. You need a magnifying glass as well as your glasses to read it, don't you? (Informal carer; pp1).

HSCPs identified that a coordinated response involving several professionals was needed to support medication management:

Well, if they're having problems taking it at the right time then I would say social services because they'd need prompting to take it. If it was because they couldn't open bottles or they were getting pills mixed up, because you, like, you might get several tablets that look the same. You know, so that would be the pharmacist because you need identification. If it was because, like with PRN medication, you would maybe need a nurse to help them identify when they needed certain drugs. (Nurse – pp12)

Some informal carers agreed with this idea and added that this support needs to take into consideration different cultural traditions and religions:

Whoever is looking after (the person with dementia) if they (clinicians) are not aware of these issues, cultural issues, religious issues and traditions…then it's a big problem…you have to build the confidence according to their traditions or religion so it plays a major part in that situation if you're not aware (of cultural and religious aspects)…South Asian people (may) have little knowledge about English. (Informal carer; pp25)

In addition, medication reviews were viewed as having a potentially positive impact, but clinicians may lack the time to conduct them adequately:

In a 20 minute appointment and looking at their medication is just one part of that…most people will probably say, “No, no, its fine, I'm taking them”. But actually, you know, I don't know how in depth we go, really. (Nurse; pp7).

Again, it is important that HSCPs coordinate any review and share relevant information between themselves to improve the support given and the treatment. Failing to do this may lead to several issues:

Although the information does come back to us through the GPs, it may not always be the type of information that we always want…that patient may not be as well adherent…and not presenting it to the doctor. (Pharmacist; pp23)

According to most of the HSCPs interviewed, medication reviews have significant potential to improve medication management but should be conducted in the patient's home so that HSCPs understand the full picture of the whole process:

That's easier isn't it when you are out there seeing them in their home. It's very difficult when people come in because you have no idea really…like I say, things can sound very chaotic but actually when you are in there, you think, “Oh, no, it's OK, it's working”. It's very difficult when you are not actually seeing it for yourself. (Nurse; pp7)

Lastly, it was highlighted that medication review should focus on decreasing the complexity of the regimen and include any formulation issues, which can be a barrier to adherence. The objective of the whole process is making the medication regimen easy to follow:

If you've got a choice of inhaler but have these twice a day…or there's one that's once a day you'd, hopefully, go for the one that's once a day one if it carries the appropriate medication. So, it's just simplifying everything…get them the best medication possible, make it simple and then they are going to use it. (Nurse; pp9)

### 3.3 The role of pharmacists

HSCPs identified the role of pharmacists in giving advice on medication. However, informal carers and people with dementia tended to focus on practical aspects of the role, such as home delivery of medication, and viewed their role in terms of the supply of medication without mentioning or indeed knowing about the clinical roles pharmacists can provide:

Well, it's got to be the doctors, hasn't it, and nurses… they're the ones, aren't they (that provide support for medication management)…rather than the pharmacists…they just provide it (medication), don't they. (Informal carer; pp1)

So how do you think pharmacists could improve support to people with dementia and their carers when it comes to managing the medication? (Question from research associate)

Yes, I mean they could give…they can sort the tablets out for them and put them in a pillbox. (Informal carer; pp3)

Non‐pharmacist HSCPs confirmed that people may not be aware of the benefits of getting advice from community pharmacists on medication, which is not the best use of the pharmacists’ skills:

Well, I think we're very lucky in this country in that we do have a community network of expert pharmacists who are under‐utilised, whose expertise is under‐utilised. (GP; pp8)

HSCPs highlighted the need for pharmacists to play a more active role in the multidisciplinary team. One way of achieving this would be having pharmacists working in GP surgeries:

The main thing is if the pharmacist is attached to the practice and to the patient that would be great…I mean, why haven't they [doctors] got such good relationships with pharmacists… here's the store of knowledge. (GP; pp8).

Additionally, all four community pharmacists argued that having access to the patient's medical records would be a starting point to improve the support they provide to people with dementia and their informal carers:

Well, I think the community pharmacists are left out of the clinical loop, the loop of communication between GPs, specialist nurses and the hospital…they don't realise the potential for the community pharmacist to be a referral, you know, to co‐ordinate…If we had access to medical records…we'd be better able to support them (i.e. the patient/carer). (Pharmacist; pp 21)

This idea was explored even further by some participants, and a nurse suggested that having a specialist designated/named pharmacist with the knowledge to educate could mean better support for medication management to people with dementia and their informal carers:

Somebody in pharmacies that oversees all dementia patients…named contact for families that if they are concerned they can contact…who was responsible for that patient's medication. (Nurse; pp7)

A further suggestion was made in terms of having community pharmacists going on home visits in order to understand how people with dementia and their informal carers are coping with their medication and help to develop strategies to address any issues:

Pharmacist that could go and visit people at home. (GP; pp15)

People (who) are alone, or, like, the carer isn't up to doing it, or feeling it's too much (Informal carer; pp4)

Pharmacists recognized that the advice and role they can provide is limited without home visits. However, the current funding model may not routinely support home medication reviews:

We do not have a commissioned service where we can do medicines use reviews at the patient's home. However, my colleagues in the neighbouring (area)…do have it…and it's shown to be a valuable service. (Pharmacist; pp23)

## 4 DISCUSSION

Medication management for people with dementia living in the community is often a very complex task and supported by a triad of the person with dementia, informal carers and various HSCPs. Informal carers have a pivotal role, but yet frequently find managing the medication of someone with dementia challenging. Indeed, even carers who stated that they had everything under control identified a ‘duty to cope’ and associated emotional burden. This echoes the findings elsewhere where informal carers identified that any failure to cope would be viewed as their fault.^26^, ^38^, ^39^ This challenge increased as the complexity of the regimen increases and if the carers lacked adequate support, again echoing previous research.^11^, ^16^, ^25^, ^26^ Limited English proficiency also increased the difficulties.^5^

The challenges associated with medication management for people with dementia, their informal carers and the wider society can have potentially serious consequences. There is an increased risk of people with dementia experiencing a medication error, as clearly seen in the accounts from both HSCPs and carers, and previously identified.^40^ Making sure that their loved one takes the right medication at the right time places a significant emotional burden on the informal carer, which is not always acknowledged by the HSCP. Additionally, if informal carers fail to cope, this has wider implications for society because further resources such as admission to residential care may be required.^21^ Other research on the challenges for informal carers in the management of Behavioural and Psychological Symptoms of Dementia (BPSD) also found a ‘sense of shame’ if the carers failed to cope and that the burden is often hidden from HSCPs.^38^, ^39^

As far as we are aware, this is the first study that has focused on the barriers to community pharmacists supporting informal carers of people with dementia. Other studies have found barriers to a more clinical role for community pharmacists in the management of BPSD^28^ and pain in people with dementia.^41^ Similar to our research, the pharmacists in these studies believed that a lack of training and multidisciplinary working and lack of access to clinical records inhibited their input.^28^, ^41^ Equally importantly, we found, like other research, that informal carers tended to view the role of pharmacists in terms of medication supply.^5^, ^26^

Other studies have identified high levels of polypharmacy with potentially inappropriate prescribing in people with dementia ranging from 22%^4^ to 81.5% of participants.^6^ We found that medication review focussing on simplifying the regimen might be helpful, particularly if delivered in the home environment. However, a systematic review found that pharmacist‐led chronic disease management increased the complexity of the medication regimen.^42^ Ultimately, it is essential that HSCPs adopt a person‐centred approach and pay attention to the challenges that informal carers and people with dementia face. This should include exploring ways to reduce any burden on informal carers associated with managing medication.

More generally, medicine optimization – defined as a ‘person‐centred approach to safe and effective medicines use, to ensure people obtain the best possible outcomes from their medicines’ – has been identified a key priority within primary care.^43^, ^44^ Further research on how to improve outcomes from medication and deliver medicine optimization is needed.^44^ Deprescribing which is considered to be ‘the process of withdrawal of inappropriate medication, supervised by a health‐care professional with the goal of managing polypharmacy and improving outcomes’ may represent a promising approach to medicine optimization.^45^ However, evidence on the impact of deprescribing in older people is equivocal and further evidence from controlled studies is required.^46^

### 4.1 Implications for clinicians and policymakers

Clinicians and policymakers need to be aware of the challenges involved and that even informal carers who do not report difficulties may be experiencing an emotional burden. Evidence‐based guidelines need to consider this burden, which is worsened as the complexity of the regimen increases. There is a potential for community pharmacists to work outside the ‘four walls’ of the community pharmacy in order to support people with dementia and their informal carers manage their medication.^47^ This ‘work without boundaries’ should include medication reviews in the patient's home, needs full access to clinical records and be coordinated with other medication optimization activity.^48^ Whilst home‐based medication reviews are occurring in some areas, this research found inconsistencies between regions. This need to review medication in the patient's own home would equally apply to practice‐based pharmacists.

### 4.2 Strengths and weaknesses

The study has a number of potential strengths. Data were triangulated from the perspectives of different HSCPs, informal carers and people with dementia. Participants were recruited from various locations and data saturation was achieved for the complete set of interviews, based on criteria from Fusch et al.^49^

Like all research, there are limitations. Findings are context‐bound to the participants and study setting, like all qualitative research.^50^ Although we believe that the testimonies from the participants were particularly rich in content, as data were obtained from face‐to‐face interviews, we cannot avoid the possibility that participants may have given socially desirable responses. Only a limited number of participants from the BME community were interviewed.

### 4.3 Future research

Further research would benefit from exploring the perspectives of a more diverse sample including people from the BME community; this study only included people from the South Asian BME community, and people with dementia living alone. A future project could also follow a cohort of people with dementia and their informal carers longitudinally to gain further insights on how the challenges change as the dementia progresses.

Ultimately, this research can inform the development of an intervention. Set within the Medical Research Council (MRC) framework, a feasibility study of a pharmacist‐led home medication review in collaboration with other HCSPs should be conducted. The intervention should be tailored to the specific needs of the person with dementia and their informal carer.^38^, ^39^

## 5 CONCLUSIONS

Medication management in people with dementia living in the community is frequently a very complex process. As dementia develops, medication management becomes the responsibility of informal carers, who frequently have little or no medical experience or knowledge. Informal carers frequently find this role challenging, particularly when the person with dementia lacks an understanding of the need for medication. This challenge was compounded by, at times, limited support from HSCPs.

Community pharmacists could potentially develop an enhanced role in supporting medication management in this area. The support should be delivered in the person's home, and the informal carer should be included in the process. It should include practical support to help them organize the medication and develop strategies to avoid medication errors, and medication review. Barriers to an expanded role include the difficulty accessing pharmacy services; more generally, this is an issue, which should receive priority in terms of funding. Informal carers may also not be aware of this potential expanded clinical role. Community pharmacists would need to develop this role within a multidisciplinary framework with access to full clinical records.

People with dementia are amongst the most vulnerable members of society with needs over and above people with no health issues. They therefore need additional support, which needs to be recognized by commissioners of services. Appropriate care pathways should be established to provide this support; community pharmacists can be a key element of these pathways.

## CONFLICTS OF INTEREST

None declared.

## ETHICS APPROVAL

Aston University Ethics Committee.

## APPENDIX 1

### Demographic details of participants

**Table:**

Interview number	Interviewee(s)	Gender	Location	Ethnicity
001	Person with dementia and Informal carer	Male (M) and Female (F)	West Midlands	White (both)
002	Informal carer	M	South West England	White
003	Person with dementia & Informal carer	M & F	West Midlands	White (both)
004	Informal carer	F	North East England	White
005	General practitioner (GP)	F	North East England	White
006	Informal carer	F	North East England	White
007	Practice nurse	F	North East England	White
008	GP	M	London	White
009	Practice nurse	F	West Midlands	White
010	Paid carer	F	West Midlands	White
011	Person with dementia	M	West Midlands	White
012	District nurse	F	West Midlands	White
013	District nurse	F	West Midlands	White
014	Person with dementia and Informal carer	M & F	West Midlands	White (both)
015	GP	F	North West England	White
016	2 Informal carers (caring for the same person)	M & F	West Midlands	White (both)
017	Paid carer	F	South East England	White
018	District/mental health nurse	F	West Midlands	White
019	GP	F	West Midlands	White
020	Paid carer	F	South East England	White
021	Community pharmacist	F	Yorkshire	White
022	Community pharmacist	M	Yorkshire	White
023	Community pharmacist	M	London	South Asian
024	Community pharmacist	F	South West England	Not known
025	Informal carer	M	Yorkshire	South Asian
026	Informal carer	F	Yorkshire	South Asian
027	Informal carer	F	Yorkshire	South Asian

### Semi‐structured Interview Schedule

1. Interview schedule for informal carers/people with dementia: A qualitative study exploring medication management in people with dementia living in the community and the potential role of the community pharmacist

*This semi‐structured interview will consist of open‐ended questions, which are split into four sections. However, these questions will depend upon how much detail the participant wants to give. Due to the sensitive nature of this topic, participants will take some direction over this interview and some questions may be missed out or expanded upon. Minor amendments may be made as the interview progresses as issues may arise that the researcher had not considered*.

#### Introduction

**Before we begin this interview, could you confirm whether you are caring for someone with dementia?**

**Thank you for taking part in this interview, I really do appreciate the time you have given. Before we begin, I want to make it clear that if you wish to skip any question(s) during the interview, or if you want to stop the interview, all you have to do is say; you do not need to give any explanation for doing so.**

Are you happy for me to begin?

Can you tell me a bit about yourself? For example, do you have family or do you work?

Can you describe what a typical day involves for you?

**The next few questions are going to be in regard to medication.**

(If people with dementia) Can you describe to me any practical issues/concerns (if any) you find with taking medication?

(If carer) Can you describe to me any practical issues/concerns (if any) you find with assisting somebody with their medication?

Can you describe to me any strategies you use to organize medication?

Prompt: From sorting your medication to taking it or assisting someone to take it; are there any particular methods/procedures you use to do this?

**In this next section, I am going to ask a few questions with regard to taking (if interviewing the person with dementia) or assisting somebody (if the carer) in taking medication. I want to remind you that these questions are to help us better our understanding of how people feel about medication. Your replies will be treated so that no one will be able to identify that these are your answers.**

(If person with dementia) Can you tell me about a time, if any, you have decided not to take your prescribed medication?

(If carer) Can you tell me about a time, if any, you have chosen to not give prescribed medication to your (whatever the relationship)?

Prompt: Do you think that people should always take their prescribed medication?

What would you describe as the benefits of taking medication?

Prompt: In your opinion, are there any benefits to not taking medication?

Can you think of any reasons why people would not take their prescribed medication?

**The next section of questions will be about your experiences with medication:**

In your opinion, does managing your medication affect your daily life?

Have you had any changes to medication over time?

If yes, can you tell me a bit about that and how it made you feel?

If no, do you think change in medication over time would have an effect on someone in your situation? If yes, in what way?

(If carer) Have you become more involved in assisting in the organization of medication?

If yes, how did that make you feel?

**We are moving onto the last section of the interview now; this section is to do with your opinions and experiences with health‐care professionals:**

Can you tell me about the experiences you have had with health‐care professionals (i.e. GPs, pharmacists, social care workers) over the past couple of years?

Prompt: Have you had any notable experiences with health‐care professionals?

Can you describe to me your feelings about the level of support you have in regard to health‐care professionals helping you with medication?

How do you think health‐care professionals could improve support to people with dementia and their carers when it comes to helping with medication management?

Prompt: Do you need support from health‐care professionals in regard to medication management? If yes, what kind of support would be helpful?

How do you feel about the future in terms of support from health‐care professionals in helping with medication?

Prompt: Do you think health‐care professionals need to be more involved in supporting people with dementia and their carers manage their medication?

Prompt: What health‐care professional do you think would be most helpful in offering this support?

Thank you

Is there anything that you would like to add or any questions you would like to go back to?

End of Interview

2. Interview schedule for health and social care professionals: A qualitative study exploring medication management in people with dementia living in the community and the potential role of the community pharmacist

*This semi‐structured interview will consist of open‐ended questions, which are split into three sections. However, these questions will depend upon how much detail the participant wants to give. Participants will take some direction over this interview and some questions may be missed out or expanded upon. Minor amendments may be made as the interview progresses as issues may arise that the researcher had not considered*.

#### Introduction

**Before we begin this interview, could you please confirm what your job title is?**

**Thank you for taking part in this interview, I really do appreciate the time you have given. Before we begin, I would like to make it clear that if you wish to skip any question(s) during the interview, or if you want to stop the interview, all you have to do is say; you do not need to give any explanation for doing so.**

Are you happy for me to begin?

Can you tell me what your current role is in regard to caring for people with dementia?

**The next few questions are going to be about your experience of reviewing and assisting medication management for dementia patients and their carers that live at home (are not in residential care).**

Can you tell me a bit about your experience of reviewing or helping people with dementia or their carers manage their medication?

From your experience and your own opinion, what would you say (if any) are the challenges in medication management for dementia patients and their carers?

Prompt: Practical challenges? How might it affect their lifestyle?

Do you feel that assisting/reviewing medication for people with dementia and their carers is a part of your role?

Prompt: Can you describe any facilitators or barriers that help or hinder you from assisting/reviewing medication?

**The next couple of questions are going to be about compliance/adherence to medication.**

Can you tell me whether you have ever experienced patients who have dementia not adhere to prescribed medication?

Prompt: If yes, can you tell me a bit about this and how did you manage this?

What do you believe could be the potential reasons for non‐adherence/non‐compliance in people with dementia/their carers?

**The last section of the interview focuses on your opinions about current practice.**

Can you tell me about your beliefs on the level of support available for people with dementia and their carers in regard to medication management?

How, in your opinion, do you think the service could improve?

Prompts: In order to make any improvements, what should take place?

In your opinion, can you describe to me the role that community pharmacy plays in medication management in people with dementia?

What do you think health‐care professionals’ role is with supporting care for medication management in people with dementia and their carers who dwell in the community?

What are your expectations of health‐care professionals’ future role in supporting people who have dementia and their carers with regard to medication management?

Do you anticipate much change happening in practice regarding this?

Thank you. Is there anything that you would like to add or any questions you would like to go back to?

End of Interview

#### Domain 1: Research team and reflexivity

**1. Which author/s conducted the interview or the focus group?**

*The interviews were conducted by Lydia Aston, Tiago Moutela or Andrea Hilton. Ian Maidment provided supervision*.

**2. What were the researcher's credentials?**

*IM and AH are both pharmacists experienced in dementia care. LA and TM are Health Psychologists in training with a Masters in Health Psychology (including a module in advanced qualitative methods)*.

**3. What was their occupation at the time of the study?**

*LA and TM were RAs specifically employed on this project. IM was a Senior Lecturer in Clinical Pharmacy, Aston University. AH was a Senior Lecturer at Hull University and a practising Community Pharmacist*.

**4. Was the researcher male or female?**

*IM and TM are male; LA and AH female. (NB – IM, LA and AH would be classed as British White; TM as Portuguese)*.

**5. What experience or training did the researcher have?**

*IM and AH are pharmacists experienced in dementia care. LA and TM have conducted numerous qualitative research projects as part of their Masters and working as RA's at Aston University*.

**6. Was a relationship established prior to study commencement?**

*LA and TM did not know any of the participants prior to study commencement. AH personally knew one of the pharmacists (given the geographical location), full informed consent was gained. They introduced the topic before the interview started and formally introduced themselves; this included explaining their current role in the project*.

**7. What did the participants know about the researcher?**

*The participants knew that IM of Aston University was leading the research and it was funded by Pharmacy Research UK and that LA and TM were research assistants and that AH was an academic from Hull University*.

**8. What characteristics were reported about the interviewer/facilitator?**

*LA and TM reported that they were research assistants conducting interviews in this area with little clinical knowledge; AH reported that she was an academic from Hull University*.

#### Domain 2: Study design

**9. What methodological orientation was stated to underpin the study?**

*An exploratory, qualitative study was conducted employing semi‐structured interviews. Data were analysed using framework analysis*.

**10. How were participants selected?**

*Informal carers were eligible if:*

- *They had provided or still provide some sort of assistance with medication management to a person who has been diagnosed with dementia and is living in the community;*
- *Do not receive any sort of payment (excluding carers’ allowance)*.

*Health and social professionals were eligible if:*

- *they had been in contact with or had been providing assistance to people with dementia*

**11. How were participants approached?**

*Participants were recruited from the Alzheimer's society, Dementia UK, local surgeries and other professional networks, and local dementia support groups (including a support group for people with dementia in the black and minority ethnic [BME] community in Yorkshire). The institutions were contacted by LA and IM via telephone and email and asked about their willingness to collaborate in advertising the study. Also, they were provided with information and details of the research, what taking part in this study would involve and the criteria individuals had to meet to be able to participate*.

*Recruitment was also conducted through snowball effect as contacts were requested to suggest other PwD, informal carers and HSCPs who might be willing to take part in this study. After their approval to collaborate in advertising this study, the contact details of IM and LA were provided and potential participants were free to contact IM and LA via email or phone to express their interest in participating. An email was then sent to all potential participants, who expressed an interest in the study, with an information sheet with details of the study and a consent form followed by a request of their availability in case they agreed in taking part*.

**12. How many participants were in the study?**

*The final sample comprised of 31 participants [eleven informal carers; four PwD, sixteen Health or Social Care Professionals (HSCPs). From the sixteen HSCPs, four were general practitioners, five were nurses (practice and district nurses), three were paid carers and four were community pharmacists]*.

**13. How many people refused to participate or dropped out?**

*None*.

**14. Where was the data collected?**

*The interviews with carers and people with dementia were conducted in the person's home. The interviews with health and social care professionals were conducted either face‐to‐face in their place of work or over the phone*.

**15. Was anyone else present besides the participants and researchers?**

*No‐one else was present except for interviews pp 025, 026 and 027 when a translator/interpreter from the same community was present. The interpreter did not hold an official qualification however they were from the same background and spoke the same language as the participants. The interpreters had been involved in previous studies where they have translated and were identified by an academic from the same community. He was confident of the accuracy of the translation and the results were consistent with other findings*.

**16. What are the important characteristics of the sample?**

*Informal carers were eligible if: they had provided or still provide some sort of assistance with medication management to a person who has been diagnosed with dementia and is living in the community and did not receive any sort of payment (excluding carers allowance)*.

*Health and social professionals were eligible if they had been in contact with or had been providing assistance to people with dementia*.

**17. Were questions, prompts, guides provided by the authors? Was it pilot tested?**

*The interview schedule consisted of open‐ended questions to avoid leading participants’ responses. Prompts were included in the schedule and were employed if required. LA and IM with support from AH developed the interview schedule. No pilot test took place*.

**18. Were repeat interviews carried out?**

*No interviews were repeated*.

**19. Did the research use audio or visual recording to collect the data?**

*A Dictaphone was used to record the interviews. Data were transcribed verbatim*.

**20. Were field notes made during and/or after the interview or focus group?**

*Additional notes were made during the interviews*.

**21. What was the duration of the interviews or focus group?**

*The length of each interview was not formally recorded. However, on discussion with LA, TM and AH, the interviews with HSCPs lasted on average approximately 20 to 30 minutes; the interviews with carers/PwD 60 to 90 minutes*.

**22. Was data saturation discussed?**

*Data saturation was reached and discussed amongst all co‐authors*.

**23. Were transcripts returned to participants for comment and/or correction?**

*Transcripts were not returned to the participants, but potential themes were discussed between all co‐authors*.

#### Domain 3: Analysis and findings

**24. How many data coders coded the data?**

*TM and IM independently reviewed the transcripts. Disagreements on the interpretation and analysis of the data were then discussed between TM and IM until consensus was achieved. A systematic cross‐comparison analysis was undertaken by TM, and reviewed by IM to identify the similarities and differences between the different participants and to develop a set of themes which represent the whole corpus of data. TM and IM then discussed and agreed the final structure of the matrix for the analysis*.

**25. Did authors provide a description of the coding tree?**

*No*.

**26. Were themes identified in advance or derived from the data?**

*Themes were derived from the data. Three key concepts were elicited from the data with subthemes within these three themes*.

**27. What software, if applicable, was used to manage the data?**

*NVIVO software was used to manage the data*.

**28. Did participants provide feedback on the findings?**

*Participants did not provide feedback*.

**29. Were participant quotations presented to illustrate the themes/findings? Was each quotation identified?**

*Extracts presented in the results section were drawn directly from the transcripts, which are recordings of the interviews*.

**30. Was there consistency between the data presented and the findings?**

*Yes there was consistency between the data presented and the findings*.

**31. Were major themes clearly presented in the findings?**

*Three key concepts were elicited from the data with subthemes within these three themes, and all three themes were discussed in the results section*.

**32. Is there a description of diverse cases or discussion of minor themes?**

*Yes, diverse cases have been discussed and the differences between the interview data from the carers and HSCPs. When relevant, minor themes were discussed*.
